# A systematic review on the epidemiology and treatment options of multiple Myeloma in Asia

**DOI:** 10.1016/j.heliyon.2024.e39698

**Published:** 2024-10-22

**Authors:** Wee-Joo Chng, Chandramouli Nagarajan, Shang-Yi Huang, Pankaj Malhotra, Yu-Yan Hwang, Vivian Blunk, Manmohan Singh, Lin Wang

**Affiliations:** a Department of Hematology-Oncology, National University Cancer Institute, Singapore; b Cancer Science Institute of Singapore, National University of Singapore, Singapore; c Department of Haematology, SingHealth Duke-NUS Blood Cancer Centre, National Cancer Centre, Singapore; d Department of Haematology, Singapore General Hospital, Singapore; e National Taiwan University Hospital, Taipei, Taiwan; f Department of Clinical Hematology and Medical Oncology, Postgraduate Institute of Medical Education and Research, Chandigarh, India; g Queen Mary Hospital, University of Hong Kong, Hong Kong, China; h Medical Affairs, Pfizer Emerging Markets, Sao Paulo, Brazil; i Medical Affairs, Pfizer Hong Kong Ltd, Hong Kong, China

**Keywords:** Newly diagnosed multiple myeloma, Relapsed/refractory multiple myeloma, Autologous stem cell transplant, Proteasome inhibitors, B-cell maturation antigen targeted chimeric antigen receptor T-cell therapy

## Abstract

Multiple myeloma (MM) accounts for almost 15 % of all neoplastic malignancies around the globe. This systematic review intends to analyse data on the treatment and management of MM in selected regions in Asia to identify and prioritize areas that need attention. A comprehensive review of original articles, published in English from 2005 to 2022, derived from the PubMed/MEDLINE database was conducted based on the Preferred Reporting Items for Systematic Reviews and Meta-Analyses (PRISMA) guidelines. There were 98 studies from select regions of Asia (China, India, Taiwan, Hong Kong, and Singapore) on newly diagnosed MM and relapsed/refractory MM. This review evaluated the trends in disease outcomes with the gradual shift in treatment regimens from doublet to triplet. Additionally, this review also explored autologous stem cell transplant outcome and anti-B-cell maturation antigen (BCMA) chimeric antigen receptor (CAR) T-cell therapy in MM patients. This is the first systematic review attempting to collect data on the utility and comparison of innovative agents and modifications in treatment regimens in the context of the Asian population. This review established that the body of evidence for the management of MM was generally of poor quality and there is a need for more versatile studies in the region. Novel and innovative drug regimens may help in combating the illness but consorted efforts by researchers, industry partners, policymakers, and the government are key factors in the long-term survival of MM patients. In the current systematic review, the authors have tried to give a comprehensive account of the available treatments, trends in MM management and prognosis for MM in Asia.

## 1 Introduction

Multiple myeloma (MM) is a progressive neoplastic B-cell malignancy of the bone marrow, representing 10%–15 % of all haematologic malignancies and 20 % of all mortality from blood and bone marrow cancers [[1], [2], [3]]. This disease is characterized by monoclonal proliferation of the plasma cells, which in turn leads to the creation of monoclonal antibodies and, ultimately, organ deterioration [3,4].

Regardless of the tremendous increase in MM diagnoses over the past 15 years, global prognosis has notably improved, likely due to novel treatment protocols and novel drugs [5]. MM's epidemiology and disease burden are poorly understood, particularly in underdeveloped nations [6]. Some MM studies show higher incidence and prevalence in Western countries (incidence rate of 3–5/100,000) than in Asia (0.5–3/100,000) [7], possibly due to genetic polymorphism [8]. MM is an incurable disease having recurrences interspersed by intervals of remission. Even with recent advancements in therapies, the majority of patients will inevitably relapse and require salvage regimens, emphasizing the continued need for novel medications throughout the disease [9]. Although MM is perceived as a single illness, it comprises numerous, distinct plasma cell clones that are cytogenetically diverse [4,5]. This diversity summons a unique and tailored treatment plan. Proteasome inhibitors (PI) bortezomib, carfilzomib and ixazomib, and immunomodulatory drugs (IMiD) such as thalidomide and lenalidomide have improved the overall survival (OS) rate. Newly approved treatment agents with different mechanisms of action include third-generation IMiD pomalidomide, histone deacetylase inhibitor panobinostat, and monoclonal antibodies such as elotuzumab, daratumumab, and isatuximab [[10], [11], [12], [13]]. Autologous stem cell transplantation (ASCT) has significantly improved survival rates over the past 20 years and is a crucial part of myeloma care [1,14].

In recent decades, new medicines have emerged. Several studies conducted in Europe and the United States have shown significant improvements in the OS rate of MM patients on a novel antimyeloma regimen followed by ASCT (5-year survival rate increased to 50%–55 %) [15]. However, the long-term prognosis of ASCT is not well documented in low-resource settings, such as in the majority of Asian countries [1,16]. There has been substantial advancement in treatment outcome trajectories in the West; however, there is a paucity of data among Asian patients [17].

The enormous lacuna of MM-related data (epidemiology, treatment, and management) in Asian countries is striking. Significant variations and disparate representation in its incidence and mortality are perceived globally, indicating inconsistencies in the availability and standard of healthcare, under-recognition of the disease, and potentially ineffective treatment approaches [18]. The treatment modalities adopted among Asian countries are in many instances outdated compared to their Western counterparts. Hence, there is a need to assess the existing research landscape on MM systematically and comprehensively to identify and prioritize areas that need attention.

This review aims to evaluate the epidemiology of MM in Asia (specifically China, India, Taiwan, Hong Kong, and Singapore), assess data from studies in select regions of Asia that have reported treatment outcomes (namely OS, progression-free survival [PFS], complete response [CR], and partial response [PR]), and review the factors affecting the treatment outcomes of MM (comorbidities, treatment regimens with/without ASCT) in Asia (in both newly diagnosed and relapsed settings). Aspects related to treatment regimens, modifications in existing regimens, patient characteristics, and factors affecting outcomes are also analyzed in this review.

## 2 Methods

### 2.1 Literature search

**Fig. 1:**
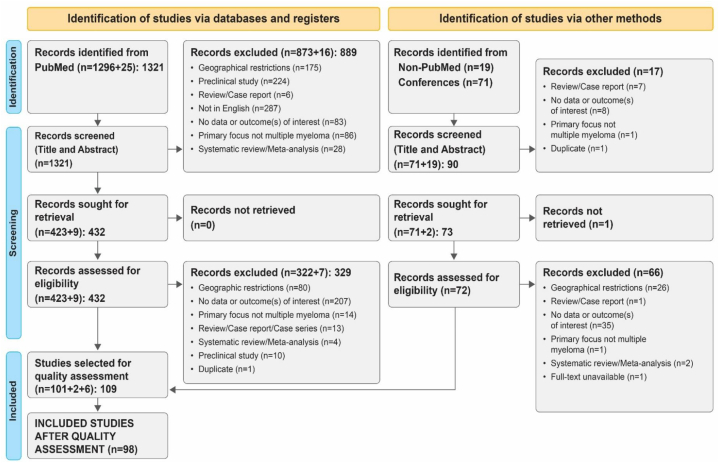
PRISMA flow diagram of study selection for the systematic review.

The literature was systematically searched using the Preferred Reporting Items for Systematic Reviews and Meta-analyses (PRISMA) reporting guidelines [19]. The search protocol is illustrated in Fig. 1. The review protocol was registered in PROSPERO, an international database of prospective systematic reviews in health and community care **(CRD42022314487)**. This review did not warrant an institutional review board approval as patient identifiers were not released. The objectives and research questions of the review were laid out at the outset to regulate the search process. Broadly, the objectives consisted of three aspects: (a) epidemiology; (b) treatment interventions (newly diagnosed, R/R, transplant-eligible, transplant-ineligible); and (c) factors affecting treatment outcomes.

Queries were designed using study-relevant search terms. The search terms included combinations of MeSH and free-text keywords using pertinent Boolean operators. The search terms used were (((multiple myeloma) AND ((incidence) OR (prevalence) OR (epidemiology)) AND ((Taiwan) OR (Hong Kong) OR (Malaysia) OR (Singapore) OR (Indonesia) OR (India) OR (China) OR (Asia))) NOT ((case report) OR (news) OR (consensus) OR (review))), (((multiple myeloma) AND ((relapse) OR (refractory)) AND ((Taiwan) OR (Hong Kong) OR (Malaysia) OR (Singapore) OR (Indonesia) OR (India) OR (China) OR (Asia))) NOT ((case report) OR (consensus) OR (review) OR (news))).

Geographical restrictions were applied to limit the search to specific regions (Taiwan, Hong Kong, China, Malaysia, Singapore, Indonesia, and India). The search strategy was designed and reviewed independently for coverage and accuracy. The review of queries was based on the Peer Review for Electronic Search Strategies (PRESS) guidelines [20].

### 2.2 Data sources

The primary source of literature for this review was PubMed/MEDLINE. The search was extended to an additional nineteen Asian journals not indexed in the aforementioned database. Original peer-reviewed articles (randomised controlled trials [RCTs] and observational studies) published in English language journals were included (from 2005 until the date of query execution – March 3, 2022). Other data sources covered original abstracts presented at the annual meetings of (1) the American Society of Hematology (ASH); (2) the American Society of Clinical Oncology (ASCO); (3) the European Society for Medical Oncology (ESMO); (4) the Asia Pacific Cancer Congress; and (5) European Hematology Association (EHA). We reviewed abstracts from these conferences corresponding to the same period as our literature search. Case series, case reports, letters to editors, preclinical studies, pharmacokinetic studies, books, book chapters, and news articles were excluded. Furthermore, articles were excepted if they were written in a language other than English or if the study was incomplete or in its nascent phase.

### 2.3 Selection criteria and screening

Predetermined inclusion/exclusion criteria were used to screen eligible articles. At the outset, only titles and abstracts were screened for inclusion. Titles and abstracts that matched the inclusion standards were selected, and their full-text versions were retrieved for review. Based on the appraisal of the full text, articles that met the screening standards were short-listed for data extraction. Three authors independently extracted data (such as authors’ names, publication date, geographical region, study population, study design, the title of the study, study objective, demographic details of the study populations, treatment regimens, outcome measures, and prognostic factors) into predefined forms on Microsoft Excel. Disagreements were reconciled by discussion. Two reviewers independently rated the quality of the included studies using the Newcastle–Ottawa scale (NOS) for case–control and cohort studies [21]. The scores were calculated according to the study outcomes, comparability, and study groups. According to the NOS, the study quality can be divided into three categories: poor (0–4), moderate (5–6), and excellent (≥7). Despite efforts to include a broad range of studies, a significant number did not meet the quality threshold, primarily due to poor reporting of selection criteria, inadequate outcome assessments, and incomplete follow-up data. A recurring limitation was the insufficient follow-up, a key factor in NOS scoring for cohort studies. Additionally, many studies failed to appropriately control for confounding variables, further lowering their quality scores. To ensure the robustness of our findings, we excluded lower-quality studies and did not perform sensitivity analyses on them. In this review, observational studies with a NOS score of 6 or greater were consigned for final inclusion.

## 3 Results

### 3.1 Summary of search

The total number of studies retrieved from (1) PubMed; (2) non-PubMed-indexed journals; and (3) Annual meetings of ASH, ASCO, EHA, and ESMO were 1411. After eliminating duplicates and studies that did not match the inclusion requirements, 109 studies were chosen for quality assessment. This review included studies with a ‘moderate and above’ score. A total of 98 studies were ultimately included in the review, of which only seven were randomized trials. All 98 studies were deemed suitable for final inclusion, as they satisfactorily met the established eligibility criteria (Fig. 1).

### 3.2 Included studies

The majority of the studies included in this systematic review (67 studies) were from China. Thirteen studies originated from Taiwan, nine from India, five from Hong Kong, and three from Singapore. Additionally, one multicenter study involved two sites in India, two sites in Singapore, and four sites in South Korea. All studies were published in international journals, with publication dates ranging from 2007 to 2021. There were 30,549 study participants in total throughout all 98 studies. The lowest and largest patient numbers in the studies were 9 and 5726, respectively [22,23]. The number of subjects who were lost to follow-up was not specified in most of the studies. The median follow-up duration across studies ranged from 6 months to 132 months [24,25]. The articles in the final collection contained epidemiological information and studies that provided comparative treatment-related outcomes.

### 3.3 Characteristics of participants

All study participants were adults, with ages ranging from 39 to 75 years [26,27]. A male preponderance was observed in MM patients from the Asian region. Most of the studies had patients with newly diagnosed multiple myeloma (NDMM) (50 studies), and only two had a mixed group of patients including NDMM and relapsed/refractory multiple myeloma (R/RMM) [28,29].

### 3.4 Incidence, prevalence, and mortality

Very few studies provided epidemiological information for the countries in focus. The incidence range per 100,000 was between 1.03 and 1.83 and the mortality per 100.000 ranged between 0.44 and 0.67. The age-standardized rates of incidence, prevalence, death, and disability-adjusted life years (DALYs) collected from the Global Health Data Exchange query tool are listed in Table 1. As per 2019 data, maximum in Hong Kong and the mortality rate is the highest in Taiwan compared to other regions. The prevalence rate and DALYs are also highest in Taiwan compared to the other regions. Although the incidence rate is high in Hong Kong, the mortality rate is comparatively low [30].

**Table 1:** Epidemiology of multiple myeloma in selected regions of Asia.

	Incidence		Prevalence		Death		DALYs	
	*2005*	*2019*	*2005*	*2019*	*2005*	*2019*	*2005*	*2019*
**China**	0.77	0.93	1.83	2.07	0.63	0.67	15.81	17.05
**India**	0.80	0.91	1.41	1.77	0.76	0.82	17.89	19.53
**Indonesia**	0.72	0.78	1.29	1.49	0.67	0.72	16.15	16.64
**Malaysia**	0.98	1.04	2.05	2.39	0.86	0.86	19.85	20.13
**Singapore**	1.34	1.35	3.77	4.27	0.99	0.93	21.52	19.72
**Taiwan**	1.52	1.67	4.23	4.95	1.13	1.20	25.60	26.85
**Hong Kong**	1.8^a^	2.0^a^	–	–	1.1^a^	0.7^a^	–	–

DALY: Disability-adjusted life years. Source: Global Health Data Exchange query tool[30].

a Hong Kong Cancer Registry.

### 3.5 Summary of treatment trends and outcomes

The identified studies evaluated the efficacy of treatment regimens considering different risk factors. Predominantly, research focused on bortezomib-based (33 studies) or thalidomide-based (24 studies) regimens or combinations (13 studies). In the relapsed or refractory (R/R) settings, studies assessing the utility of carfilzomib, daratumumab, circularly permuted TRAIL (CPT) – a recombinant human tumour necrosis factor (TNF)-related apoptosis-inducing ligand, and chimeric antigen receptor (CAR) T-cell therapy are discussed. The current review had 20 studies reporting PR, 29 studies reporting on CR, 35 studies reporting on PFS, and 32 studies reporting on OS. The lowest median PFS of 9.63 months was observed in a study using a thalidomide plus dexamethasone regimen among the 22 NDMM studies that reported PFS [31], whereas the patients who had undergone ASCT had the highest PFS of 73.8 months, as reported across 29 studies [32]. Conventional chemotherapy followed by ASCT had the least OS (17 months), while growth factors and antimicrobial prophylaxis after ASCT demonstrated the highest OS (138.3 months) among the 14 newly diagnosed cases with ASCT. Patients achieving CR or very good partial response (VGPR) after ASCT had longer OS (more than 15 months) and PFS (more than 10 months). Among the 29 studies on patients with R/RMM, the highest reported PFS was 54.7 months, observed in a study where 69 % of patients had undergone ASCT. In contrast, the lowest PFS of 5.5 months was reported in a study using CPT in combination with thalidomide and dexamethasone as the treatment regimen [33,34]. The lowest OS (10 months) used a 3-weekly daratumumab with immunomodulatory agents and dexamethasone (dara-IMiD-dex) regimen [34]. An OS of 70.4 months was observed when administering high-dose consolidation therapy (HDT) followed by ASCT [35].

A comprehensive summary of the treatment regimen and treatment-related outcomes are mentioned in Table 2, and a summary of the evidence for a few key questions that were raised in the treatment and management of MM (based on the author's discretion) is mentioned in Table 3.

**Table 2:** Summary of treatment-related outcomes.

Variables	Treatment regimen	Outcomes
*Newly diagnosed multiple myeloma (NDMM)*		
**Complete response (%)**	•Bortezomib or combinations of bortezomib in different concentrations [[36], [37], [38], [39], [40], [41], [42], [43], [44], [45]] •6 cycles of dtZ regimen three times a week [46] •Vincristine–cyclophosphamide–melphalan or mitoxantrone–prednisone in combination with thalidomide [25] •A salvage regimen of oral cyclophosphamide at 50 mg/day and oral prednisone at 15 mg/day was used [47] •Hydroxyurea, cytarabine, busulfan, cyclophosphamide, and semustine. Prophylaxis–cyclosporin, until 6 months after transplantation [26]	3.33–72.7 18 19.73 66.7 72.7
**Partial response (%)**	•Bortezomib or combinations of bortezomib [[36], [37], [38], [39], [40], [41], [42]] •G-CSF 5, antimicrobial prophylaxis, and ASCT (with maintenance therapy for 2 years) [31] •Oral cyclophosphamide and oral prednisone [47] •6 cycles of dtZ regimen three times a week [46] •Cyclophosphamide, melphalan or mitoxantrone, and prednisone in combination with thalidomide [25]	2.6–66.7 12 66.7 36.4 16.7
**Progression-free survival (months)**	•Thalidomide and dexamethasone regimen [31] •Growth factors G-CSF 5 μg/kg before ASCT. Antimicrobial (sulfamethoxazole, acyclovir, fluconazole, and trimethoprim) prophylaxis after ASCT [32]	9.63 73.8
**Overall survival (months)**	•Traditional chemotherapy (different combinations of vincristine, doxorubicin, dexamethasone, cyclophosphamide, etoposide, cisplatin, and melphalan) and new drugs (bortezomib and carfilzomib, as well as immunomodulators such as thalidomide and lenalidomide). ASCT was performed after the innovative agent-based regimens. The study participants also had severe RI [48] •ASCT (7 months median time from diagnosis to transplant). Growth factors G-CSF 5 μg/kg. Antimicrobial agents (sulfamethoxazole, acyclovir, fluconazole, and trimethoprim), prophylaxis after ASCT [31]	17 128.3
Relapsed or refractory multiple myeloma (R/RMM)		
**Complete response (%)**	•Continuous low-dose oral cyclophosphamide–prednisone administration [27] •Ixazomib plus lenalidomide–dexamethasone [49] •Carfilzomib and dexamethasone [50] •Low-dose lenalidomide and dexamethasone combination treatment [51] •PAD regimen [52] •Carfilzomib–dexamethasone–daratumumab [53] •Long-term thalidomide [54] •3-weekly daratumumab–lenalidomide [34] •ASCT [55] •CAR-T-cell therapy [[56], [57], [58], [59], [60], [61]]	3.7 5 6.5 12.5 24 45.7 50 54 44 57–86
**Partial response (%)**	•Three daily doses of fludarabine and one dose of cyclophosphamide before CAR-T-cell infusion. Humanized anti-CD19 CAR-T cells and anti-BCMA CAR-T cells were infused [58] •LCAR-B38M CAR-T cells were injected in three separate infusions [56] •3-weekly Dara-IMiD-dex regimen [34] •Long-term, high-dose thalidomide [54] •Carfilzomib and dexamethasone [50] •PAD regimen [52] •Combination of low-dose lenalidomide with dexamethasone [51] •Oral cyclophosphamide–prednisone at low doses [27] •Ixazomib plus lenalidomide–dexamethasone [49]	14 14 15.4 17 17.9 33 34 48 51
**Progression-free survival (months)**	•CPT combined with thalidomide and dexamethasone [33] •ASCT. They were also treated with dara-IMiD-dex [34]	5.5 54.7
**Overall survival (months)**	•The study employed a 3-weekly dara-IMiD-dex regimen [34] • The OS rate was high in research that used HDT followed by ASCT. Patients who had better treatment responses before ASCT had better PFS and OS than those who did not [35]	10 70.4

ASCT: Autologous stem cell transplantation; BCMA: B-cell maturation antigen; CPT: Circularly permuted trial; CAR-T cell: Chimeric antigen receptor T cell; dara-IMiD-dex: Daratumumab with immunomodulatory agents and dexamethasone; dtZ regimen: Low-dose dexamethasone-thalidomide-zoledronic acid; G-CSF: Granulocyte colony–stimulating factor; HDT: High-dose consolidation treatment; OS: Overall survival; PAD: Modified bortezomib–, adriamycin, and dexamethasone; PFS: Progression-free survival; RI: Renal impairment.

**Table 3:** Summary of the evidence for key questions in the management of multiple myeloma.

Question	Conclusion	Evidence	References
**Is chemotherapy an ideal treatment modality?**	High-dose chemotherapy followed by ASCT is helpful in tumour management. Bortezomib-based chemotherapy and vincristine–cyclophosphamide–melphalan or mitoxantrone–prednisone is beneficial in MM patients with RI. A combination of partial tumour excision and chemotherapy appears to be a viable treatment for MM spinal cord compression.	Based on four studies from India (two studies) and China (two studies)	[1,25,62,63]
**Is there a successful salvage therapy once the first-line therapy has failed?**	A viable alternative for people with multiple myeloma who have significant concomitant conditions or a history of recurring infections linked to traditional chemotherapy is low-dose cyclophosphamide and prednisone. In transplant-eligible patients, a staged approach using VTD is a good salvage option	Based on two studies from Hong Kong and China	[47,64]
**Is ASCT beneficial in R/RMM?**	Patients with pre-transplant treatment and frontline use of bortezomib-based induction before ASCT had better prognoses after ASCT	Based on two studies from Hong Kong and Taiwan	[35,55]
**Are allogenic (BCMA)-CAR-T-cells are better than autologous CAR-T cells in R/RMM?**	Patients who received autologous CAR-T cells had better PFS and OS rates than those who received allogeneic CAR-T cells.	Based on a Chinese study	[57]
**Is thalidomide an option after transplant failure for R/RMM patients?**	Thalidomide is safe, efficient, and practical for long-term use. Large-scale studies are required to support its use as a maintenance treatment. Patients with R/RMM who took thalidomide alone also found it to be effective and well-tolerated. When taken with dexamethasone, thalidomide was more thrombogenic than other medications.	Based on six studies from India, Taiwan, and China	[24,33,54,[65], [66], [67]]

ASCT: Autologous stem cell transplantation; BCMA: B-cell maturation antigen; CAR-T cell: Chimeric antigen receptor T cell; MM: Multiple myeloma; OS: Overall survival; PFS: Progression-free survival; RI: Renal impairment; R/RMM: Relapsed or refractory multiple myeloma; VTD: Bortezomib–thalidomide–dexamethasone.

### 3.6 Treatment and management - NDMM

This review encompassed 50 studies on NDMM. The proteasome inhibitor bortezomib emerged as a promising novel agent for NDMM in Asia. Chen J et al. and Chen X et al. explored bortezomib's impact on patients with renal impairment (RI) [40,48]. They observed higher CR rates (33.3 % vs. 3.33 %) and longer median OS (15.0 months vs. 6.0 months) with bortezomib versus non-bortezomib treatment, indicating its efficacy. Additionally, the overall renal response rate in bortezomib-based regimens was significantly higher than that in non-bortezomib based regimens. Hence indicating that bortezomib-based regimens may be the preferred treatment in patients with severe RI [40,48].

A study conducted on Chinese patients revealed that the patients with light-chain MM had an aggressive disease course, and bortezomib significantly improved their outcomes [68]. Studies indicate that regimen modifications can mitigate drug toxicity while sustaining efficacy. For instance, subcutaneous bortezomib surpasses intravenous delivery [43]. Chim et al. used a staged strategy to treat MM in a trial conducted in Hong Kong, where vincristine–adriamycin–dexamethasone (VAD)-chemosensitive patients underwent direct autologous-hematopoietic stem cell transplantation (HSCT), whereas patients with less chemosensitivity to VAD, defined as <75 % “M” protein reduction, received salvage therapy with VTD (bortezomib–thalidomide–dexamethasone) before autologous HSCT. The survival rates paralleled bortezomib-based induction therapies, confining bortezomib-based salvage to around half the patients, without adverse treatment impact. The negative prognostic impact of inadequate chemosensitivity may have been eliminated by this approach [64]. A clinical study conducted on Chinese patients with low doses of thalidomide and dexamethasone elicited a favourable clinical response with a manageable toxicity profile [32].

When compared to the PAD (bortezomib–adriamycin–dexamethasone) regimen, the VCd (bortezomib–cyclophosphamide–dexamethasone) regimen had higher therapeutic benefit and a predictable safety profile in elderly patients [69]. Another comparison of the lenalidomide–adriamycin–dexamethasone (RAD) and PAD regimens revealed that RAD swiftly improved patients' quality of life and reduced doctors' occupational stress. Nevertheless, RAD induction should be restricted to a maximum of four cycles to prevent permanent harm to hematopoietic stem cells [45]. Studies with triplet drug regimens fared better than doublet drug regimens. The BTD (bortezomib–dexamethasone with thalidomide) regimen had an overall response rate (ORR) of 97 % compared to single or double regimens using bortezomib (bortezomib and thalidomide combination had only 26 % ORR among NDMM patients) [39,70]. Another multicentered study from China also prescribed a triplet regimen of ixazomib-lenalidomide-dexamethasone (IRd). This regimen also had high ORR (95.3 %), VGPR (66 %) and CR (30 %) [71]. A triplet VTD regimen of intravenous bortezomib had an acceptable 1-year OS of 72 % [38] and another triplet regimen of lenalidomide, thalidomide, and bortezomib-based induction regimen among Indian patients had a 3-year OS of 80 % [72].

Twenty studies in this review had patients with NDMM divided into comparison groups to understand the difference in treatment outcomes based on treatment regimens. Findings from the comparisons of interventions are presented in this section in Table 4.

**Table 4:** Summary of comparisons of treatment regimens identified from the literature search.

Regimen 1	Regimen 2	Study conclusion	References	
**TBD**	T-VAD	Both regimens were effective in treating patients with NDMM.	[44]	
**Low-dose thalidomide regimen**	High-dose thalidomide regimen	The incidence of grade 3 or higher adverse events was considerably higher in the high-dose group than in the low-dose group.	[32]	
**Bortezomib regimen**	Non-bortezomib regimen	Light-chain MM patients had more aggressive disease courses and worse outcomes, which could be improved with bortezomib treatment.	[68]	
**PAD**	CBd	When compared to the PAD regimen, elderly patients treated with CBd demonstrated higher therapy advantages and a predictable safety profile.	[69]	
**Bortezomib-containing regimen**	Thalidomide-containing regimen	Patients above the age of 75 with extramedullary plasmacytoma fared poorly. Bortezomib-containing regimens had a greater CR. In terms of survival outcomes, no substantial improvement was noticed.	[73]	
**Thalidomide-based regimen**	Bortezomib-based regimen	Even with bortezomib-based therapy, patients with del (12p) had considerably worse PFS and OS. PFS and OS were improved in patients without del (12p13) after bortezomib-based therapy vs. thalidomide-based therapy. Bortezomib-based therapy did not improve the poor survival of del patients (12p13).	[74]	
**Bortezomib-containing regimen**	Non-bortezomib regimen	Combination chemotherapy based on bortezomib can enhance the prognosis of NDMM in individuals with RI and should be regarded as first-line therapy.	[75]	
**Bortezomib-based regimen**	Thalidomide-based regimen	In the thalidomide-based group, there was a substantial difference in survival across the three ISS stages, but not between ISS stages I and II in the bortezomib-based group. The data show that bortezomib may have the capacity to partially offset the negative effect of risk variables on survival, particularly at a later stage of the ISS system.	[76]	
**Conventional VTD**	Improved VTD	The revised VTD regimen, which switched bortezomib from intravenous to subcutaneous administration, had noninferior efficacy to the regular VTD regimen, as well as a better safety profile and fewer side events.	[38]	
**Conventional bortezomib**	Modified bortezomib (increased dose once weekly)	Increased-dose, weekly bortezomib-based combination therapies did not perform worse than standard treatments in terms of response and survival benefit, but they did have a reduced rate of peripheral neuropathy.	[28]	
**Subcutaneous group**	Intravenous group	Subcutaneous bortezomib has been linked to improved tolerance; however, intravenous treatment resulted in a faster and deeper response.	[43]	
**Bortezomib standard therapy**	Bortezomib weekly regimen	The once-weekly bortezomib regimen was as effective as normal therapy, although the incidence of thrombocytopenia was reduced when compared to conventional therapy.	[77]	
**High-dose (1.6 mg/m**^**2**^**) bortezomib regimen**	Low-dose (1.3 mg/m^2^) bortezomib regimen	High-dose bortezomib as an induction regimen resulted in a higher CR rate, particularly in individuals 65 years and above or with R-ISS stage III and is feasible for youthful and high-risk patients.	[36]	
**Thalidomide group**	Bortezomib group	The survival advantage acquired by the t(14; undefined) group in the bortezomib-based group was substantially greater than the t(11; 14) and t(4; 14)/t(14; 16) groups. Notably, t(14; undefined) was discovered to be an independent predictor of longer OS.	[78]	
**Lenalidomide group**	Non-lenalidomide group	In both the single-hit and multi-hit groups, the ORR of the VRD group was considerably higher than that of the non-VRD group. In terms of ASCT, tandem-ASCT improved the 2-year PFS and OS of multi-hit MM considerably.	[79]	
**RAD**	PAD	RAD induction exhibited comparable efficacy to PAD and a considerably superior safety profile, as well as enhanced patient quality of life and reduced occupational stress for doctors.	[45]	
**Bortezomib with thalidomide regimen**	Thalidomide or non-bortezomib with thalidomide regimens	Bortezomib-containing regimens were more effective than thalidomide as first-line therapy, albeit at the expense of additional outpatient visits and higher total expenses.	[80]	
**Chinese herbal medicine**	Non-Chinese herbal medicine	The results indicated that patients with MM could benefit from Chinese herbal medicine treatment, potentially improving survival rates in Taiwan.	[81]	
**Non-first-line bortezomib**	First-line bortezomib	The PFS of patients receiving first-line bortezomib treatment with transplantation differed significantly.	[23]	
**VAD-chemosensitive regimen**	Bortezomib/thalidomide in less chemosensitive regimen	The staged method demonstrated a cost-effective use of expensive targeted drugs while maintaining a high CR and OS.	[64]	

ASCT: Autologous stem cell transplantation; CBd: Cyclophosphamide–bortezomib–low-dose dexamethasone; CR: Complete response; ISS: International Staging System; MM: Multiple myeloma; Non-V/T: Non-bortezomib or thalidomide; ORR: Overall response rate; OS: Overall survival; PAD: Bortezomib–doxorubicin–dexamethasone; PFS: Progression-free survival; RAD: Lenalidomide–doxorubicin–dexamethasone; RI: Renal impairment; R-ISS: Revised-International Staging System; TBD: Thalidomide-bortezomib–dexamethasone; T-VAD: Thalidomide combined with vincristine–doxorubicin–dexamethasone; VAD: Vincristine–doxorubicin–dexamethasone; VRD: Bortezomib–lenalidomide–dexamethasone; VTD: Bortezomib–thalidomide–dexamethasone.

### 3.7 Treatment and management - R/RMM

There were only a few studies (29 studies) that investigated the use of daratumumab, carfilzomib, and CAR-T cells in patients with R/RMM. The combination of daratumumab with carfilzomib and dexamethasone showed promising results in the phase III CANDOR trial [53]. In a post hoc subgroup analysis of Asian patients from this study, efficacy and tolerability were found to be similar to those in the total treated population, suggesting that it is feasible to use this combination in Asian patients without any added risks of serious adverse events [53]. However, it should be noted that the cohort size was small.

Combinations of lenalidomide with low-dose dexamethasone and low-dose lenalidomide with dexamethasone were well tolerated by patients [51,82,83]. It also resulted in prolonged PFS and improved response duration. The combination of CPT and thalidomide demonstrated encouraging results in clinical trials for patients with R/RMM [24,33,66]. However, this treatment should be used with caution as it is a new type of recombinant therapy. The incidence of venous thromboembolism was lower among the Asian thalidomide-treated patients compared with their Western counterparts [65]. Two studies conducted in China revealed that a modified treatment regimen of combining CPT with dexamethasone and thalidomide significantly improved the response rate in patients with R/RMM [33,66]. Furthermore, the combination of CPT, which is a human Apo2L/TRAIL mutant, with dexamethasone and thalidomide was considered a potential new treatment for patients with MM. It prolonged the OS (21.8 months) and PFS (5.5 months) of the patients and demonstrated an improved ORR [33].

Studies assessing the potential of anti-BCMA–directed CAR-T cell therapy for patients with R/RMM found it to be safe and effective [61,84,85]. Deng et al. examined its viability in R/RMM with extramedullary disease (EMD) and concluded that anti-BCMA CAR-T cell therapy allows for a period of remission, which can be maintained by combining other modalities of treatment like radiation and HSCT [84]. In another study, patients who received autologous CAR-T cell therapy had longer PFS and OS (more than 2 years) than those who received allogeneic CAR-T cell therapy. The disparity in the impact between autologous and allogeneic CART infusion was also demonstrated by the maximum alteration in serum myeloma protein concentrations [57]. Studies using anti-BCMA CAR-T cell therapy had reasonably higher ORR - (98.3 % and 87.5 %), CR - 79.2 %, PFS - 50.2 % and OS - 78 % [59,61]. On the other hand, the CR rate was 24.7 % from bortezomib only-based regimen on patients with R/RMM in a real-world study [86].

### 3.8 Critical factors affecting treatment outcomes

Factors affecting the prognosis/treatment outcomes of MM are established in Table 5.

**Table 5:** Factors affecting the prognosis/treatment outcomes of MM.

Factors affecting treatment outcomes	Regimen	Treatment outcome/prognosis	Reference
**Patients with IGH deletion + ve**	Bortezomib and/or thalidomide-based chemotherapy	Patients with deletion showed improved ORR to PAD induction therapy.	[42]
**Higher decorin∗ levels in bone marrow plasma**	Chemo-based: VAD, MP; novel agents-based: BTD, BTD and cyclophosphamide	H-DCN was linked to improved treatment results and a longer PFS.	[70]
**Cytogenetic abnormalities in MM**	Bortezomib and non-bortezomib group	Del (17p), t(4; 14), and 1q21 gain are all independent risk factors for MM patients. Patients with these anomalies are more likely to relapse early.	[87]
**TNF promoter polymorphisms**	Thalidomide and dexamethasone	TNF-alpha238 GA + AA genotypes were significantly linked with improved PFS and OS.	[29]
**Heterogeneous chromosome 12p deletion**	Thalidomide and bortezomib groups	Patients with the deletion had a low survival rate when treated with either bortezomib or thalidomide.	[74]
**PHD finger protein 19/PHF 19 (also known as polycomb‐like protein 3 [PCL3]) expression in multiple myeloma**	BDT, BD, Allo-HSCT	Increased PHF19 expression is linked with poor induction therapy response and a negative prognosis of MM.	[88]
**Pre-treatment neutrophil/lymphocyte ratio**	Conventional and bortezomib-based chemotherapy	Increased NLR is a poor predictive factor in elderly patients and the advanced stages of MM.	[89]
**Early monoclonal protein decline pattern**	Regular bortezomib-based chemotherapy followed by ASCT	Those with pattern B showed greater PFS and OS than patients with pattern A or C.	[90]
**CTLA-4 polymorphisms**	PAD, VCD, VTD	CTLA-4 rs733618 GG decreased PFS and OS in MM patients.	[91]
**The effect of type 2 diabetes on the survival of MM patients**	Chemotherapy/VAD/thalidomide/bortezomib/HDT and HSCT/bisphosphonates	Pre-existing diabetes – higher mortality risk when compared to non-diabetic peers.	[92]
**RI in MM patients**	(1) Bortezomib-containing regimens (2) Novel agent-based regimen followed by ASCT (3) (4) BCMA (CAR)-T cell therapyBortezomib in combination with dexamethasone (5) Lenalidomide with low-dose dexamethasone	(1) Prognosis was better in the bortezomib groups than the non-bortezomib groups. (2) Severe RI-adverse prognostic factor for survival in NDMM. In individuals with severe RI, bortezomib-based regimens may be the preferable treatment. (3) CAR-T-cell therapy used to treat R/RMM may improve renal function. (4) Bortezomib in combination with dexamethasone is a safe and efficient treatment for NDMM (with RI). (5) Patients with no/mild RI had a longer PFS and OS compared to those with moderate or severe RI.	[40,75] [48] [93] [94] [83]
**Hypercalcemia**	Bortezomib or the thalidomide-based induction followed by ASCT on two groups (patients with hypercalcemia and patients without hypercalcemia)	The OS was double-fold in the non-hypercalcemia patients compared to the patients with hypercalcemia.	[95]
**Elevated LDH**	Bortezomib-based induction and ASCT regimen	A poor prognostic factor for MM	[96]
**Presence of EMD**	(1) Bortezomib and thalidomide groups (2) A 3-weekly daratumumab regimen in R/RMM followed by ASCT (3) Humanized BCMA (CAR)-T cell therapy in R/RMM (4) Glutathione combined with mecobalamin to treat PN in MM (5) Bortezomib treatment in light-chain MM	(1) EMD has a poorer outcome and CR is higher in the bortezomib-containing regimen. (2) Information on EMD is unavailable. (3) CRS and ICANS were significantly greater in EMD patients. Patients with EMD have a poor prognosis. (4) Mortality risk of MM patients with EMD (2.373) was greater than that of non-EMD MM patients. (5) EMD was more common among the light-chain MM compared to other subtypes.	[73] [34] [61,84,85] [97] [68]

Allo-HSCT: Allogeneic hematopoietic stem cell transplantation; ASCT: Autologous stem cell transplantation; BDT: bortezomib–dexamethasone–thalidomide; BCMA: Anti-B-cell maturation antigen; BD: bortezomib–dexamethasone; BTD: Bortezomib–dexamethasone with thalidomide; CAR: Chimeric antigen receptor; CR: Complete response; CRS: Cytokine release syndrome; Cy: Cyclophosphamide; CTLA-4: Cytotoxic T-lymphocyte-associated protein 4; DM: Diabetes mellitus; EMD: Extramedullary disease; H-DCN: High decorin; HDT: High-dose therapy; HSCT: Hematopoietic stem cell therapy; ICANS: Immune effector cell-associated neurotoxic syndrome; IGH: Immunoglobulin heavy chain gene; MM: Multiple myeloma; MP: Melphalan and prednisolone; NDMM: Newly diagnosed multiple myeloma; NLR: Neutrophil/lymphocyte ratio; OS: Overall survival; PAD: Bortezomib–doxorubicin–dexamethasone; PFS: Progression-free survival; PN: Peripheral neuropathy; RI: Renal impairment; R/RMM: Relapsed and refractory multiple myeloma; TNF: Tumor necrosis factor; VAD: Vincristine–doxorubicin–dexamethasone.

∗ It is the only instance, where decorin is mentioned in the review. We have presented the findings from a study (included in this review) which compared decorin with other treatment regimens.

### 3.9 Studies with transplant-related findings

All 32 studies in this review that contained transplants as a part of treatment protocol showed positive overall outcomes. Studies using ASCT soon after diagnosis have a better prognosis. In almost all the studies (where the transplant was possible), the transplanted group had improved PFS, OS, and ORR [16,23,31,48,78,79,98,99]. Although ASCT has greatly improved the prognosis of patients with NDMM, there are still factors that can affect the initiation of the transplant process, especially in Asia.

In a retrospective data analysis from a tertiary care facility in India, the most common reasons for not receiving a transplant were financial constraints and concern about complications [16]. Two Indian studies and a Taiwanese study have demonstrated significant variations in outcomes between patients who received ASCT and those who did not [16,31,98]. Patients with ASCT had improved OS, PFS, and ORR. Early post-ASCT recurrence and suboptimal post-transplant response were suggested to be more important determinants of PFS and OS than pretransplant factors such as the International Staging System (ISS) or cytogenetics [17]. The inclusion of such aspects in the risk stratification systems to rationalize therapy is proposed by the Singapore Multiple Myeloma Working Group [17]. The prognosis of high-risk cytogenetic abnormalities such as multi-hit MM remains poor. The use of lenalidomide–bortezomib–dexamethasone (VRD) induction regimen followed by tandem-ASCT has been proposed as an effective treatment modality for multi-hit myeloma compared to single-hit ASCT [79].

### 3.10 Distinctive adverse events among the included studies

Several treatment-emergent adverse events were identified from the studies. These adverse events were either the result of a comparison of regimens or other factors that impact outcomes. The assessments made in the studies are intended to reduce toxicity and/or achieve a useful risk–benefit profile.

In the current review, the adverse events observed across most of the studies were similar. Studies with triplet regimens had more severe adverse events to some extent. The most common adverse events observed in the triplet regimens were gastrointestinal symptoms (diarrhoea, constipation, nausea, and vomiting), anaemia, neutropenia, leukopenia, thrombocytopenia, lymphocytopenia, and cytokine release syndrome (CRS).

Additionally, across the triplet regimen studies, patients with renal failure and EMDs had more acute adverse events [39,84]. The critical treatment-related adverse events are summarised in Table 6.

**Table 6:** Critical treatment-emergent adverse events highlighted in group-wise comparisons.

Group 1	Group 2	Adverse event of interest	Reference
**Severe RI**	Non-RI	Lenalidomide with low-dose dexamethasone therapy was associated with a higher prevalence of grade 3–4 neutropenia, anaemia, and sleeplessness in patients with severe RI.	[83]
**Increased dose, weekly bortezomib**	Standard bortezomib regimen	A weekly bortezomib regimen reduces the risk of peripheral neuropathy and thrombocytopenia incidence.	[28,77]
**Subcutaneous bortezomib**	Intravenous bortezomib	Grade 3 or higher peripheral neuropathy was lower in the subcutaneous bortezomib group.	[43]
**Conventional VTD**	Improved VTD (subcutaneous)	When compared to traditional lenalidomide, low-dose dexamethasone therapy was associated with a higher prevalence of grade 3–4 neutropenia, anaemia, and sleeplessness in patients with severe RI. VTD, grade 3 and above adverse events were 50 % less common in the enhanced VTD group (80 %).	[38]
**Low-dose thalidomide (mean sustained dose, 180.4 mg)**	High-dose thalidomide (mean sustained dose, 292.9 mg)	Incidences of adverse events of grade 3 and above were higher in the high-dose group.	[32]
**PAD**	RAD	Infections, peripheral neuropathy, and gastrointestinal problems were more common in the PAD group, whereas leukopenia and rashes were more common in the RAD group.	[45]
**P1AD (bortezomib, 1.3 mg/m**^**2**^**)**	P2AD (bortezomib, 1.0 mg/m^2^)	When compared to the P1AD group, toxic symptoms such as thrombocytopenia, peripheral neuropathy, and gastrointestinal response were significantly inhibited in the P2AD group.	[52]
**Patients receiving glutathione and mecobalamin**	Patients not receiving glutathione and mecobalamin	Significantly reduced incidence and severity of peripheral neuropathy in patients receiving glutathione and mecobalamin.	[97]
**CPT + thalidomide**	Placebo + thalidomide	Reduced neutrophil counts (26.8 % vs. 26.6 %), pneumonia (25 % vs. 23.7 %), and hyperglycemia were all noted in the CPT group (21 % vs. 12.2 %).	[33]
**CPT (5 mg/kg); CPT (8 mg/kg); CPT (10 mg/kg)**	N/A	Neutropenia, anaemia, alanine transaminase, and leukopenia were the most common grade 3–4 treatment-related side events.	[24]
**KdD**	Kd	In the KdD and Kd arms, 95.7 % and 90.0 % of patients had grade 3 or higher treatment-emergent adverse events, respectively.	[53]
**Patients with EMD**	Patient without EMD	Patients with EMD had greater grades of CRS and ICANS after receiving anti-BCMA CAR-T cell treatment.	[84]
**CLTA-4 SNP rs4553808** **GG + GA**	CLTA-4 SNP rs4553808 AA	When compared to the CLTA-4 SNP rs4553808 AA group, the incidence of non-haematologic grade 3–4 adverse events were considerably higher in the CLTA-4 SNP rs4553808 GG + GA group when treated with a bortezomib-based regimen.	[91]

Anti-BMCA CAR-T: Anti-B-cell maturation antigen chimeric antigen receptor T-cell therapy; CLTA-4: Cytotoxic T-lymphocyte-associated protein-4; CPT: Circularly permuted trail; CRS: Cytokine release syndrome; EMD: Extramedullary disease; ICANS: Immune effector cell-associated neurotoxicity syndrome; Kd: Carfilzomib–dexamethasone; KdD: Carfilzomib–dexamethasone–daratumumab; PAD: Bortezomib–doxorubicin–dexamethasone; R/R: Relapsed/refractory; RAD: Lenalidomide–doxorubicin–dexamethasone; RI: Renal impairment; SNP: Single-nucleotide polymorphism; VTD: Bortezomib–thalidomide–dexamethasone.

## 4 Discussion

This systematic review aimed to collect and analyse data on the treatment regimens, modifications, and management strategies for MM in Asia. The authors observed a lower MM-related incidence and mortality in Asia compared to the Western world. However, Asian MM treatment outcomes were somewhat inferior, possibly due to delayed diagnosis, constrained resources affecting care, and limited access to clinical trials and emerging therapies [7]. For example, the number of MM cases surged in China, Taiwan, and North Korea, rising from 4760 cases in 1990 to 17,218 cases in 2016. This increase was accompanied by a significant rise in age-specific incidence rates. The observed discrepancies may be attributed to inferior diagnostic capabilities and limited healthcare resources in low-income countries, which hinder early detection and effective management of the disease. Nevertheless, these variations do not necessarily reflect biological differences in MM across regions [100].

The volume of studies eligible for the review was notably limited. High-quality studies were mostly from China, and a few studies were from the Indian subcontinent or the Far East Asian countries (Singapore, Taiwan, and Hong Kong). Yanamandra and Malhotra highlighted the rise in the incidence of MM in China, North Korea, and Taiwan (262 % increase from 1990 to 2016) [101]. This uneven distribution reflects the lack of comprehensive epidemiological studies and the absence of national myeloma registries in some countries, such as India. While 34 of the 98 studies reviewed included transplant-related regimens, research consistently shows that ASCT significantly improves OS from 2–3 years to 8–10 years. Notably, India has the lowest ASCT rates globally, indicating an unmet need for accessible transplant services in this region [101,102].

The scarcity of clinical trials across Asia, particularly RCTs, reflects regulatory barriers, logistical challenges, and limited pharmaceutical industry investment [103]. This dearth of trial data complicates the ability to draw strong conclusions about treatment strategies. Nonetheless, this review identified various treatment regimens with promising outcomes. For example, some studies reported prolonged survival when monoclonal protein reduction was achieved using bortezomib-based chemotherapy followed by ASCT and maintenance therapy [90]. The OS among the pattern B patients in this study was 117 months (the longest OS across the studies in this review) [104]. However, variabilities in OS and PFS between studies employing similar treatments highlight the impact of small sample sizes and short follow-up periods. Several studies demonstrated that triple-drug regimens improve response rates, leading to higher remission rates, ORR, PFS, and OS in both NDMM and R/RMM [37,46,53,105].

Triple-drug regimens demonstrated superior efficacy compared with double-drug regimens, improving outcomes such as ORR, PFS, OS, VGPR, and CR. However, these regimens are often associated with increased grade 3–4 adverse events and may not be suitable for all patients. The selection of triplet regimens should consider individual patient characteristics, disease attributes, and cost constraints in certain countries [106,107]. Furthermore, Huang et al., revealed that using a bortezomib-only regimen among patients with R/RMM who were previously prescribed thalidomide and prednisone, the CR and VGPR were in the ranges of only 1.1%–3.8 % throughout the 8 cycles [108]. This yet again reiterates the inferior response rate of a single-drug chemotherapy regimen.

In this review, immunotherapy such as anti-BCMA CAR-T cell therapy, has shown great prognosis in patients with R/RMM [61,84,85]. (CAR) T-cell therapy has shown significant promise in R/RMM, extending survival and remission rates even in nonresponsive patients [109]. The most common side effects of anti-BCMA CAR T-cell therapy are CRS, immune effector cell–associated neurotoxicity syndrome (ICANS), tumour lysis syndrome, and neurotoxicity [109,110]. Studies have also reported an increase in the incidence of infections post anti-BCMA CAR T-cell therapy (23 %–63 %) [110]. A single-centre retrospective analysis found prevalent bacterial, viral, and fungal infections in 29 of 55 (53 %) patients, 1 year post-therapy, primarily affecting upper or lower respiratory systems. This was the largest study to date to assess the infectious complications after BCMA CAR-T [110]. Despite these risks, the benefits of CAR T-cell therapy often outweigh the potential complications, making it a viable option for select patients [109].

The aggressive form of MM -EMD, largely affects the course of the disease as well as the treatment outcome. Globally, the incidence of EMD increased from 6.5 % in 2005 to 23.7 % in 2014 [111], and patients with EMD in this review demonstrated shorter PFS compared with those without EMD [84]. Although intensive chemotherapy may offer some respite, the OS and prognosis often remain unfavorable [111,112]. The limited data available on EMD treatment outcomes highlight the need for prospective studies to evaluate therapeutic strategies in these patients. The heterogeneity of MM imparts variability in clinical and survival outcomes. Optimum risk stratification is crucial to predicting and designing an ideal treatment strategy. Response-based changes in treatment strategy need further investigation. This review concludes that there is a requisite for further studies with patient-level information in the future.

Limitations of the review: Country names without variations were used in the search string, which may have restricted the scope of the search and led to the omission of relevant studies. However, the overall impact on the findings is expected to be minimal, as the search strategy still captured a substantial amount of data pertinent to the research objectives. Nonetheless, this limitation is acknowledged to ensure transparency in the methodology. Additionally, there was significant variability in study designs, treatment regimens, and outcome measures across the included studies. This heterogeneity made it challenging to generalize the findings to a broader population. Furthermore, although there was substantial evidence on the treatment and management of MM, there was very little detailed comprehensive information concerning prognostic indicators such as ORR, OS, PFS, CR, PR, and VGPR.

Some of the outcome-specific limitations we could identify in this review were: (i) potential bias due to mostly non-RCT studies; (ii) one of the outcome indicators in this study (ORR) is a physician-determined subjective assessment; hence, bias in the extracted data is inevitable; (iii) varying median follow-up times. Short follow-up periods are not sufficient to capture long-term treatment outcomes, particularly for therapies like ASCT and CAR-T cell therapy, where extended follow-up is crucial to assess survival benefits. The extension of the period could have positively influenced the direction of the outcome.

## 5 Conclusion and recommendations

Through a comprehensive analysis of the literature, we were able to identify a lacuna in the quality of clinical trials and prospective studies conducted in the region. Additionally, the data across the studies were non-homogeneous. This warrants further research into the treatment landscape of MM in Asia with an emphasis on the treatment outcomes of various drug regimens. The surge in the incidence of MM in Asia along with established ethnic susceptibility to the treatment outcome and prognosis of MM might warrant a revamp of the treatment protocols currently being followed in many countries and regions.

Financial constraints in availing timely and appropriate care were a recurring theme observed among the studies included in this review. Due to the aforementioned reasons, the management of MM can be challenging, necessitating the development of cost-effective diagnostic methods and novel therapeutics. Therefore, to effectively tackle the unmet needs pertaining to the diagnosis and management of MM, the authors have enumerated several recommendations.

### 5.1 Recommendations

1. Development of Novel, Cost-Effective Therapies: Governments and industry partners must collaborate to ensure the availability and accessibility of innovative drug regimens proven to improve survival rates and quality of life for patients with MM in Asia.
2. Collaborative Research Initiatives: Establishing multicenter partnerships between research institutions, hospitals, and international organizations can facilitate resource-sharing, improve data quality, and enhance MM management in the region.
3. Patient Education and Awareness: Improving healthcare professional and public awareness is crucial for early diagnosis and better disease management. Patient education programs can empower patients to make informed decisions, leading to improved adherence and treatment outcomes.
4. Epidemiological Studies: Extensive research on the incidence, prevalence, and risk factors of MM in Asia is required to inform and provide treatment strategies and understand the disease burden in the region.
5. Creation of National Myeloma Registries: Establishing regional or national registries can help systematically gather and analyse data on patients with MM, enabling better tracking of disease outcomes and the identification of treatment gaps.
6. Encouragement of Clinical Trial Participation: Asian patients should be encouraged to participate in clinical trials to enhance population diversity and ensure that treatment recommendations are applicable across different ethnicities.
7. Palliative Care Integration: The establishment of MM-specific palliative care units integrated into treatment protocols can improve the quality of life for patients, particularly those with advanced or refractory disease.

In conclusion, while significant progress has been made in MM management, there remains an urgent need for further research, policy development, and resource allocation to address the unique challenges faced by patients with MM in Asia.

## CRediT authorship contribution statement

**Wee-Joo Chng:** Writing – review & editing, Resources, Formal analysis, Data curation, Conceptualization. **Chandramouli Nagarajan:** Writing – review & editing, Resources, Data curation, Conceptualization. **Shang-Yi Huang:** Writing – review & editing, Methodology, Data curation, Conceptualization. **Pankaj Malhotra:** Writing – review & editing, Resources, Methodology, Data curation, Conceptualization. **Yu-Yan Hwang:** Writing – review & editing, Data curation, Conceptualization. **Vivian Blunk:** Writing – review & editing, Validation, Data curation, Conceptualization. **Manmohan Singh:** Writing – review & editing, Validation, Data curation, Conceptualization. **Lin Wang:** Writing – review & editing, Validation, Data curation, Conceptualization.

## Note on PRISMA checklist

Our review is designed as a narrative synthesis, emphasizing a qualitative analysis of the included studies rather than a quantitative meta-analysis. Hence, certain items were not applicable in the study. However, we would like to assure you that, while certain items were deemed not applicable, their exclusion does not compromise the integrity of our study. We welcome any additional inquiries and are prepared to provide further clarification if needed.

## Data and code availability statement

The data referenced in this article are included within the text. Additional data generated and/or analyzed during the study are available from the corresponding author upon reasonable request. However, access to certain data may be restricted due to privacy or ethical considerations.

## Funding

This work was supported by 10.13039/100004319Pfizer.

## Declaration of competing interest

The authors declare the following financial interests/personal relationships which may be considered as potential competing interests:Chandramouli Nagarajan reports financial support was provided by Janssen. Chandramouli Nagarajan reports a relationship with Janssen that includes: board membership and speaking and lecture fees. Chandramouli Nagarajan reports a relationship with Amgen that includes: speaking and lecture fees. Chandramouli Nagarajan reports a relationship with AZ that includes: board membership and speaking and lecture fees. Chandramouli Nagarajan reports a relationship with Sanofi that includes: board membership. Chandramouli Nagarajan reports a relationship with Pfizer that includes: board membership. Chandramouli Nagarajan reports a relationship with BMS that includes: board membership. Vivian Blunk reports a relationship with Pfizer that includes: employment and equity or stocks. Manmohan Singh reports a relationship with Pfizer that includes: employment and equity or stocks. Lin Wang reports a relationship with Pfizer that includes: employment. If there are other authors, they declare that they have no known competing financial interests or personal relationships that could have appeared to influence the work reported in this paper.
